# The Investigation of Ni-Doped SrFeO_3−δ_ Perovskite for a Symmetrical Electrode in Proton Ceramic Fuel Cells

**DOI:** 10.3390/ma18071460

**Published:** 2025-03-25

**Authors:** Jiajia Cui, Yueyue Sun, Chaofan Yin, Hao Wang, Zhengrong Liu, Zilin Zhou, Kai Wu, Jun Zhou

**Affiliations:** 1 School of Materials Science and Engineering, Xi’an University of Technology, Xi’an 710048, China; 2 Center of Nanomaterials for Renewable Energy, State Key Laboratory of Electrical Insulation and Power Equipment, Xi’an Jiaotong University, Xi’an 710049, China; sunyue98@stu.xjtu.edu.cn (Y.S.); yinchaofan@stu.xjtu.edu.cn (C.Y.); liuzhengrong@stu.xjtu.edu.cn (Z.L.); linda623532@stu.xjtu.edu.cn (Z.Z.); wukai@mail.xjtu.edu.cn (K.W.); zhoujun@mail.xjtu.edu.cn (J.Z.); 3 Hengtong Group, Suzhou 215200, China; wanghao@hengtonggroup.com.cn

**Keywords:** proton ceramic fuel cells, symmetrical electrode, thermal matching, boosted electrochemical performance

## Abstract

The development of symmetrical solid oxide fuel cells with identical cathode and anode is beneficial for thermal matching and reducing the cost. Herein, proton-conducting electrolyte and novel high catalytic activity electrode material for symmetrical solid oxide fuel cells are proposed. Ni-doping at the B-site of (Sr_0.8_Ce_0.2_)_0.95_FeO_3−δ_ (SCF) indicates reduced cell edge lengths, cell volume, and a more porous honeycomb structure. The B-site elements in oxide tend to have a high oxidation state via Ni-doping. Simple doping modification in SCF causes better thermal matching between the electrode and electrolyte and form more oxygen vacancies at the operating temperature. At the anode side, Ni-doping improves the stability of the symmetric electrode in reducing the atmosphere. The polarization resistance of symmetrical cells for new electrode material is half of the original both in oxidation and reduction atmosphere, which indicates boosted electrochemical performance for the cathode and anode. At the same time, Ni-doping reduces the impedance activation energy of the anode reaction in symmetric cells. The output performance of the cell is 210.4 mW·cm^−2^ at 750 °C and the thickness of the electrolyte is 400 μm, achieving a highly efficient symmetrical electrode in proton ceramic fuel cells. The new finding of materials provides a novel high efficiency symmetrical electrode and proposes guidance for the improvement of solid oxide fuel cells at a reduced temperature.

## 1. Introduction

The rapid development of society depends on energy conversion devices. Solid oxide fuel cells (SOFCs) have been considered one of the most promising devices for direct conversion of chemical energy in fuel into electricity with high energy efficiency and low emissions [1,2,3,4]. However, the high operating temperature (800–1000 °C) of SOFCs leads to the mismatch of components, long start-up times, strict material requirements, and high prices [5,6,7,8]. It seriously hinders the commercial application of SOFCs. The traditional SOFC is based on an oxygen ion conducting electrolyte, which demands a high operating temperature [9,10,11,12]. The main reason is that the thermo-activation reactions of the oxygen ion conducting electrolyte requires high temperatures [13,14,15,16]. Currently, a proton-conducting electrolyte replaces the oxygen ion conducting electrolyte for greater proton mobility to design proton ceramic fuel cells (PCFCs), which effectively reduced the operating temperature [17,18,19,20]. Therefore, current research is focusing on finding high catalytic and thermal matching electrodes for PCFCs.

The perovskites in ABO_3_ type are favorable candidates for electrodes in PCFCs [21,22,23]. Generally, the A-site is large cations such as rare-Earth or alkaline-Earth elements and the B-site is transition elements with smaller ionic radius. The ABO_3_ perovskites possess a special framework of [BO_6_] octahedra and structural stability under a reducing or oxidizing atmosphere, which are employed as symmetrical electrode materials for SOFCs [24,25,26]. Perovskite oxide SrFeO_3−δ_ exhibits excellent redox properties in oxygen exchange applications, including oxygen separation and chemical cycling for oxygen production. However, its kinetics are insufficient at lower temperatures [27,28,29,30]. The oxidation reaction rate may be limited by diffusion in the bulk, while the reduction reaction rate is limited by surface reactions. By doping minor Ca at A-site, Sr_0.93_Ca_0.07_Fe_0.9_Co_0.1_O_3−δ_ indicates an increased oxidation rate by four times [31]. Yao et al. employed Ta^5+^ and Mo^6+^ co-doped at the B-site of SrFeO_3−δ_ perovskite as cathodes for intermediate temperature SOFC, which indicates the increased number of oxygen vacancies in the material and better electrochemical performance of the cell [32]. The W-doped SrFeO_3−δ_ of SrFe_0.8_W_0.2_O_3−δ_ perovskite oxide prepared by Liu et al. was applied as an electrode material for symmetric solid oxide fuel cells. W-doping not only stabilizes the cubic perovskite structure of SrFeO_3−δ_ but also increases its resistance to reducing atmospheres [33]. Ce-doping at the A-site of SrFeO_3−δ_ increases its structural stability in a reducing atmosphere, and SSOFCs with Ce/Ru co-doped exhibit excellent electrochemical performance [34].

In this regard, a proton-conducting electrolyte instead of the traditional oxygen ion conducting electrolyte is employed, seeking a symmetrical electrode material with high catalytic activity. The novel symmetrical electrode material of Ni-doping at B-site of (Sr_0.8_Ce_0.2_)_0.95_FeO_3−δ_ is designed with a porous honeycomb structure, which is beneficial for gas transport and reaction. Via Ni-doping, the B-site elements tend to have a high oxidation state, which can form more oxygen vacancies for oxygen transport during oxygen reduction reaction at the cathode side. Furthermore, Ni-doping is expected to be realized as a better matched thermal expansion coefficient between the electrode and electrolyte. In an attempt to explore the process of electrochemical reaction, the polarization resistance and output performance of the cell is investigated to provide a novel high efficiency symmetrical electrode material and guidance for achieving low- temperature PCFCs.

## 2. Experimental and Analysis

### 2.1. Materials Synthesis

**Figure 1:**
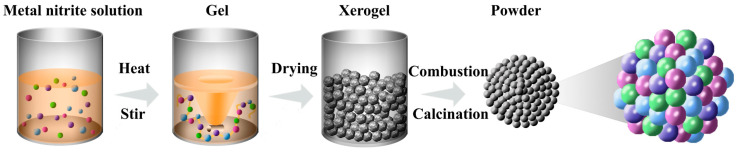
The schematic diagram of the sol–gel method to prepare SCF and SCFN.

The target compositions of the symmetrical electrode materials (Sr_0.8_Ce_0.2_)_0.95_FeO_3−δ_ (SCF) and (Sr_0.8_Ce_0.2_)_0.95_Fe_0.9_Ni_0.1_O_3−δ_ (SCFN) powders were synthesized by the sol–gel method of citric acid-nitrate. Stoichiometric amounts of Sr(NO_3_)_2_ (99.5% Aladdin, Wuhan, China), Ce(NO_3_)_3_·6H_2_O (99.0% Aladdin, China), Fe(NO_3_)_3_·9H_2_O (98.5% Aladdin, China), and Ni(NO_3_)_2_·6H_2_O (99.5% Sinopharm Group, Shanghai, China) were dissolved in deionized water for the compositions of SCF and SCFN. Citric acid with the molar ratio of the total metal in 2:1 was added into the solution. Then, the solution was continuously stirred and heated to form a gelatinous state. The xerogel was sintered at 600 °C for the burning of citric acid and calcined at 950 °C for 10 h in air to yield the final perovskites. The specific experimental process is shown in Figure 1.

### 2.2. Cell Fabrication

BaZr_0.1_Ce_0.7_Y_0.2_O_3_ (BZCY) powder for the electrolyte was synthesized by the solid reaction method. First, stoichiometric amounts of BaCO_3_ (99.9% Aladdin, China), ZrO_2_ (99.99% Aladdin, China), CeO_2_ (99.99% Aladdin, China), and Y_2_O_3_ (99.99% Sinopharm Group, China) were mixed well by ball milling, and the mixture was sintered at 1200 °C for 10 h. The pre-sintering powder was pressed into a disk under a pressure of 10 MPa and calcined at 1450 °C for 10 h to obtain the dense electrolyte disk. To fabricate the symmetric cells, the powders of SCF and SCFN were mixed uniformity with terpilenol and turpentine and then screen-printed on both sides of a BZCY disk and sintered at 950 °C for 4 h; the current collector was formed by silver paste sintered on both sides of the electrolyte. The sample bars used for the dilatometer test are prepared by dry pressing and a solid reaction method at 1300 °C.

### 2.3. Characterization

**Figure 2:**
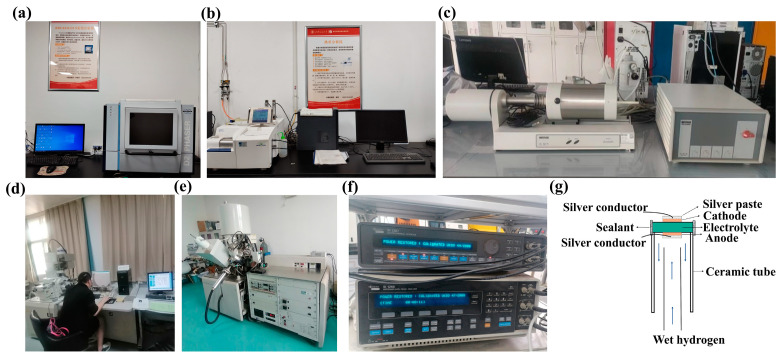
Experimental facilities. (**a**) XRD; (**b**) TG; (**c**) DIL; (**d**) SEM; (**e**) XPS; (**f**) electrochemical workstation; (**g**) schematic diagram of cell test device.

X-ray diffraction (XRD, Berlin, Germany, Bruker D2 PHASER) patterns of the prepared samples were conducted in the 2θ range of 20–80° operated at a step of 0.02° using Cu tube, as well as the mixture of electrodes and electrolyte sintered at 950 °C in air and 5%H_2_. Thermogravimetric (TGA, Mettler Toledo, Greifensee, Switzerland) analyses were performed from room temperature to 800 °C in oxygen with a heating rate of 5 °C·min^−1^. Thermal expansion coefficients (TECs) of the symmetrical electrodes and the electrolyte were measured by dilatometer (DIL 402C, Netzsch, Selb, Germany) to evaluate the compatibility between the electrolyte and electrode. The morphologies of the electrode powders and the interface of the cell were performed by scanning electron microscopy (SEM, KEYENCE VE-9800, Osaka, Japan). X-ray photoelectron spectroscopy (XPS, Thermo Scientific K-Alpha, Waltham, MA, USA) was performed to determine the different valences of the elements in the samples before and after reduction. The relevant experimental instruments are shown in Figure 2a–e. Electrochemical impedance spectroscopy (EIS) is one of the most commonly used tools for studying electrochemical systems. The impedance spectrum can be analyzed by the equivalent circuit, that is, the circuit diagram composed of some electrical components is fitted to the behavior of the electrochemical system and a series of parameters such as polarization resistance are obtained. Figure 2f shows Solartron 1260–1287, an electrochemical workstation used for testing electrochemistry in this paper. It characterizes AC impedance spectra under different atmospheres and ambient temperatures and uses Z-view software (Z-view 3.0a) attached to the instrument to analyze and fit the collected impedance spectra data. The electrochemical impedance spectra of the symmetrical cell are typically measured by an electrochemical workstation in the frequency range from 0.1 Hz to 1 MHz under an applied amplitude of 10 mV. The cell performance was tested by an electrochemical workstation by using air as the oxidant and wet H_2_ as the fuel. The symmetrical cell is fixed at one end of the high temperature ceramic tube, sealed with a high temperature inorganic adhesive, and the sealed battery is placed in the air for curing for at least 24 h, as shown in Figure 2g. Run the high temperature furnace at a heating rate of 2 °C·min^−1^ to slowly heat up to the cell test temperature to prevent the cracking of high temperature inorganic glue. After ensuring the air tightness of the test device, wet H_2_ is used as the fuel gas for the anode side, and static air is used as the oxidizing agent for the cathode side. The electrochemical workstation is used to test the output performance of the cell. The output voltage (*V*) and current (*I*) of the cell are measured. The power density *P* can be calculated by the Formula (1) as follows:

(1) P=VI/S

where *P* is the output power density of a single cell (mW·cm^−2^); *I* is the output current (mA); *V* is the working voltage (V); and S is the effective working area (cm^2^) of the cell.

## 3. Result and Discussion

**Figure 3:**
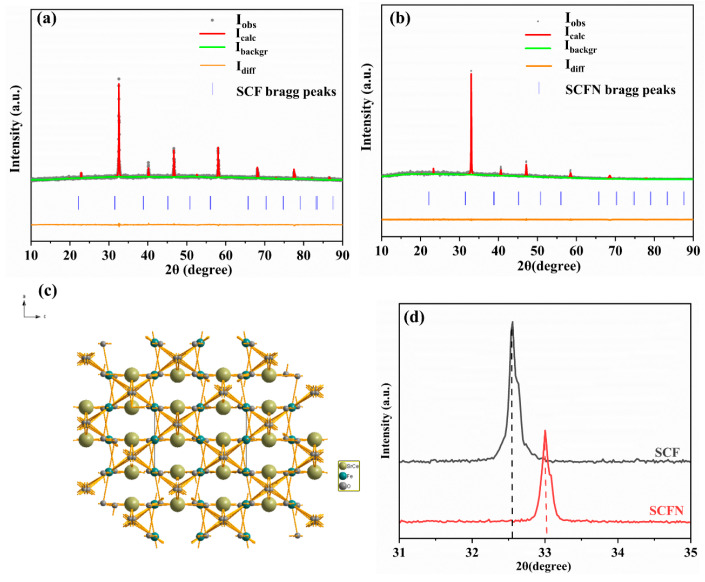
The XRD refinements of SCF (**a**) and SCFN (**b**). The crystal structure of the electrode (**c**). (**d**) The enlarged view of SCF and SCFN from 31° to 35°.

Figure 3a,b displays the X-ray diffraction pattern and the full profile refinements of SFC and SCFN, indicating orthorhombic structure (Figure 3c) sintering in air at 950 °C for 10 h. That is, Fe ions at the B-site were successfully replaced by Ni ions in (Sr_0.8_Ce_0.2_)_0.95_FeO_3−δ_. The main peaks of the XRD pattern can be indexed by SrFeO_3−x_ (JCPDS No.34-0641), belonging to space group *Pbnm* (62). The cell parameters, cell volume, and other information obtained by XRD refinement are listed in Table 1. Via Ni-doping at the B-site, the cell edge lengths a, b, and c of the ABO_3_ type perovskite were reduced, and the cell volume decreased from 235.9 Å^3^ to 234.7 Å^3^. Furthermore, the XRD peak of SCF at around 32.6° is slightly shifted to the right by 33.0° for SCFN with Ni-doping shown in Figure 3d. As ionic radii of Ni are smaller than those of Fe, the results are in accordance with the Vegard’s rule of the lattice volume. This further confirms that the ion valence state at the B-site is demonstrated in the XPS analysis.

**Figure 4:**
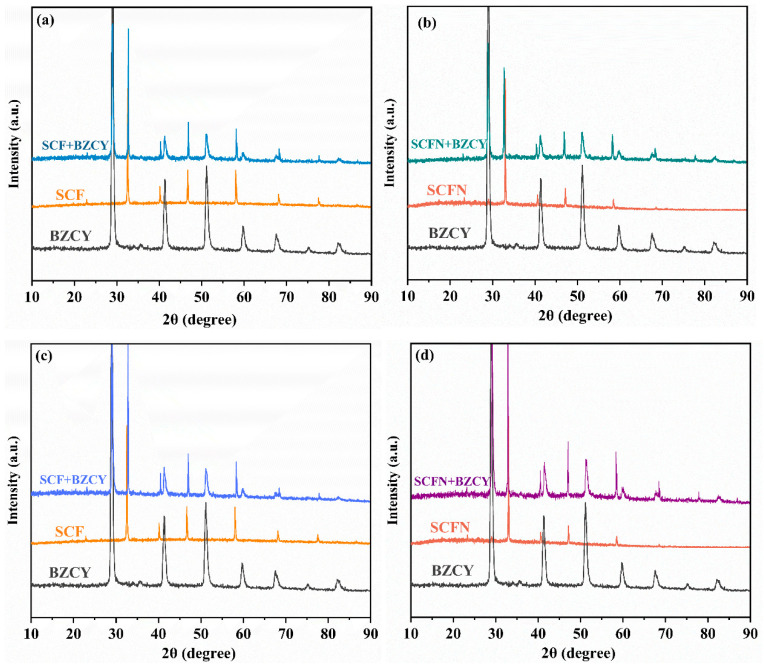
Chemical compatibility between electrode and electrolyte. (**a**,**b**) In air for 6 h; (**c**,**d**) in 5%H_2_ for 6 h.

The chemical compatibility between the electrode and electrolyte in the oxidizing/reducing atmosphere is analyzed in Figure 4. The XRD patterns of the symmetrical electrode material, electrolyte and the mixture of the electrode and the electrolyte sintered at 950 °C in air for 6 h are shown in Figure 4a,b. The XRD of the symmetrical electrode, electrolyte and the mixture of the electrode and the electrolyte sintered in 5%H_2_ at 950 °C for 6 h are shown in Figure 4c,d, respectively. The results show that, both for SCF and SCFN, the XRD patterns of the mixture undergoing high temperatures are the superposition of the diffraction peaks of the cathode and electrolyte. Obviously, no new impurity peaks appear. This indicates excellent chemical compatibility between the cathodes and electrolyte BZCY.

**Figure 5:**
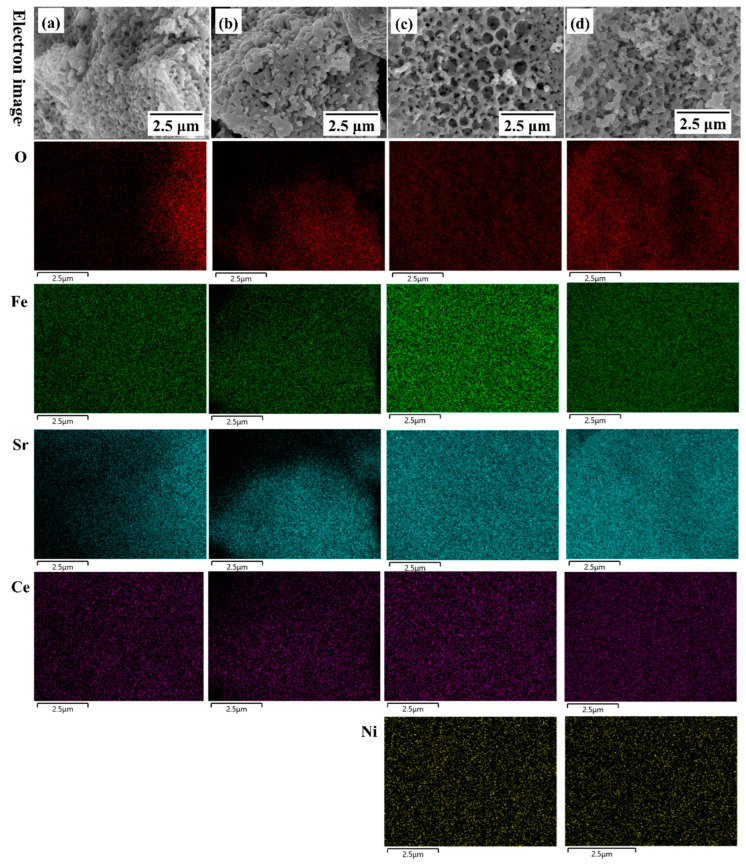
SEM and EDS mappings for SCF before (**a**) and after reduction reaction (**b**); SCFN before (**c**) and after 2 h reduction reaction at 800 °C in 5%H_2_ (**d**).

The SEM and Energy Dispersive X-ray Spectroscopy (EDS) mappings for SCF and SCFN before and after the reduction reaction are shown in Figure 5, where, in general, the elements are distributed uniformly in the electrode. Via Ni-doping at the B-site, the electrode material presents a more porous honeycomb structure, which is conducive to the gas transport during the reaction process. As shown in Table 2, the atomic percents for SCF is 53.3%, 24.0%, 18.0%, and 7.3% for O, Fe, Sr, and Ce before reduction. The reduction reaction resulted in a significant decrease in the surface elements of O, Sr, and Ce, and a significant increase in Fe. For SCFN, the atomic percents are 53.7%, 21.1%, 18.3%, 4.5%, and 2.5% for O, Fe, Sr, Ce, and Ni before reduction. The reduction reaction resulted in a slight decrease in O, a slight increase in Fe, and the atomic ratios of the other elements barely changed. This indicates that Ni-doping not only accelerates the gas transport of the redox reaction process, but also facilitates the stability of the electrode material in the reducing atmosphere.

**Figure 6:**
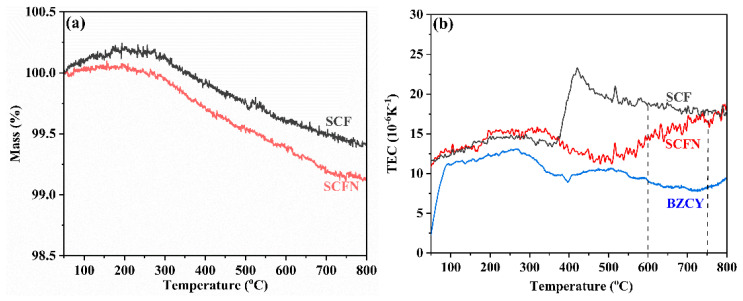
TGA curves (**a**) and thermal expansion curves (**b**) of electrode and electrolyte as function of temperature.

Thermal compatibility is a critical issue between the electrode and electrolyte. It can be evaluated by the material thermal expansion coefficient (TEC). Figure 6a shows the instantaneous TEC of the electrode and electrolyte in air. This indicates that the TEC of BZCY fluctuates in the range of 8.0–13.0 × 10^−6^ K^−1^. The TEC of SCFN is increased with rising temperature at the operating temperature (600–750 °C) in the range of 14.3–16.8 × 10^−6^ K^−1^. Compared with SCF, the difference in TEC between SCFN and BZCY is relatively small, implying that Ni-doping SCF at the B-site improves the thermal match with the BZCY electrolyte. According to TG analysis in Figure 6b, SCF and SCFN indicate a minor increase in mass from room temperature to 200 °C, which is derived from the adsorption of reaction gas O_2_ by porous cathode materials. Above 200 °C, both SCF and SCFN exhibit accelerated mass reduction, with mass percents of 99.4% and 99.1%, respectively. As the temperature rises, the B-site metal oxides are reduced and lattice oxygen escapes, forming oxygen vacancies. The formation of oxygen vacancies accelerates the diffusion and migration of oxygen at the cathode side, thereby enhancing the electrocatalytic activity. Compared with SCF, Ni-doping at the B-site leads to an increase in weight loss, which is beneficial for the formation of oxygen vacancies.

**Figure 7:**
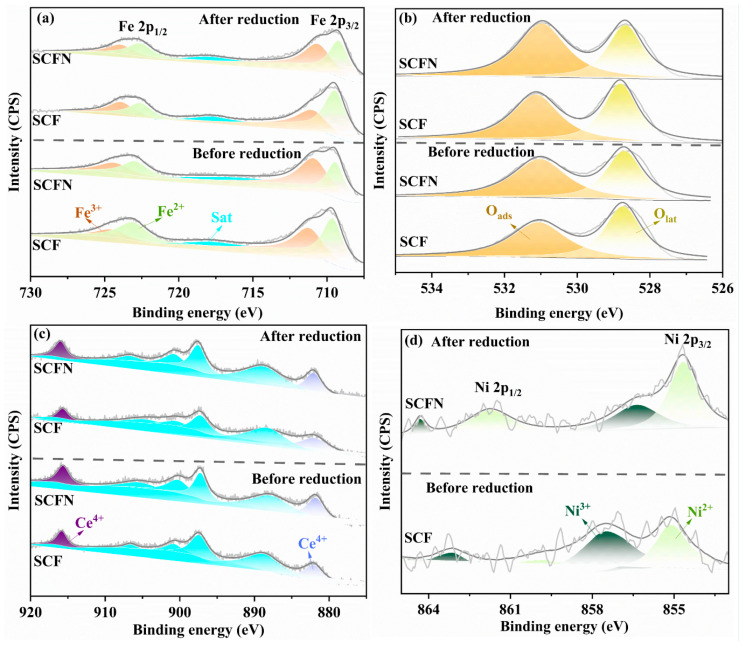
XPS spectra of SCF and SCFN samples before and after 2 h reduction at 800 °C in 5%H_2_ in terms of (**a**) Fe 2p, (**b**) O 1s, (**c**) Ce 3d, and (**d**) Ni 2p, respectively.

The XPS spectra of the as-prepared and reduced samples of SCF and SCFN were analyzed to study the chemical composition and valence states of the elements. As shown in Figure 7a, there are two distinct peaks in Fe 2p, Fe 2p_1/2_ and Fe 2p_3/2_, which contain Fe^2+^ and Fe^3+^. For Fe 2p_1/2_, peaks with higher binding energies are assigned to Fe^3+^, while peaks with lower binding energies belong to Fe^2+^. For Fe 2p_3/2_, the peak with high binding energy belongs to Fe^3+^, while the peak with low binding energy is assigned to Fe^2+^. Figure 7b shows the XPS of O 1s. The peak with higher binding energy is approximately 531 eV, which represents lattice oxygen (O_lat_) bound to metal atoms, while the relatively lower peak represents surface adsorbed oxygen (O_ads_) at approximately 529 eV. The characteristic peak at 916 eV could be related to the presence of Ce^4+^, while the low energy one at 882 eV could be related to the presence of Ce^3+^ in Figure 7c. There are two peaks in Ni 2p for SCFN in Figure 7d, Ni 2p_1/2_ and Ni 2p_3/2_. For Ni 2p_1/2_, the peak with the higher binding energy is assigned to Ni^3+^, while the peak with the lower binding energy belongs to Ni^2+^. For Ni 2p_3/2_, the peak with the high binding energy belongs to Ni^3+^, while the peak with the low binding energy is assigned to Ni^2+^.

The specific valence states of the elements are shown in Table 3. It indicates that the ions tend achieve to high valence states via Ni-doping, where the content of Fe^3+^and Ce^4+^ increases, as well as O_lat_. During high temperature operation of the cell, the reduction in B-site metal oxides and the escape of lattice oxygen lead to the formation of oxygen vacancies. The presence of oxygen vacancies is beneficial for increasing the migration rate of oxygen in the cathode material, thereby enhancing the electrochemical activity of the material. Ni-doping causes the oxidation state of the B-site elements to tend towards high oxidation states, making them prone to reduction reactions. Moreover, it increases the lattice oxygen content of the material, which can efficiently improve the electrochemical reaction activity of the cathode side. Compared with the XPS results of the reduced materials, in general, the reduction reaction causes the elements to tend towards a lower valence state. Ni-doping reduces the reduction in high valence elements and lattice oxygen in the material, which helps to improve the stability of the material during anodic reactions. Moreover, for O 1s, the reduction reaction tends to reduce O_ads_ on the surface rather than O_lat_.

**Figure 8:**
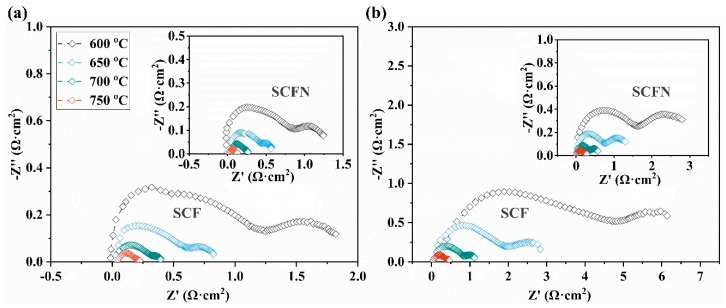
Comparison of electrochemical impedance for SCF and SCFN electrodes in air (**a**) and 5%H_2_ (**b**).

The study on electrochemical impedance performance of the symmetrical electrode was conducted with the electrolyte of BZCY, which is shown in Figure 8a,b. In general, the impedance decreases as the temperature increases. In air, the polarization resistance (*R*_p_) of SCF and SCFN is 0.22 and 0.13 Ω cm^2^ at 750 °C. In 5%H_2_, the *R*_p_ of SCF and SCFN is 0.50 and 0.28 Ω cm^2^ at 750 °C. Compared with SCF, the *R*_p_ of SCFN is reduced for both the operated anode and cathode, which is derived from Ni-doping SCF at the B-site and induced the generation of new catalysts. The specific electrochemical impedance parameters are displayed in Table 4. The results suggest that Ni-doping SCF at the B-site not only improves the thermal match with the electrolyte but also optimized electrochemical reactions. In general, the Ce/Ni co-doped (Sr_0.8_Ce_0.2_)_0.95_Fe_0.9_Ni_0.1_O_3−δ_ would be a promising symmetrical electrode for PCFCs.

**Figure 9:**
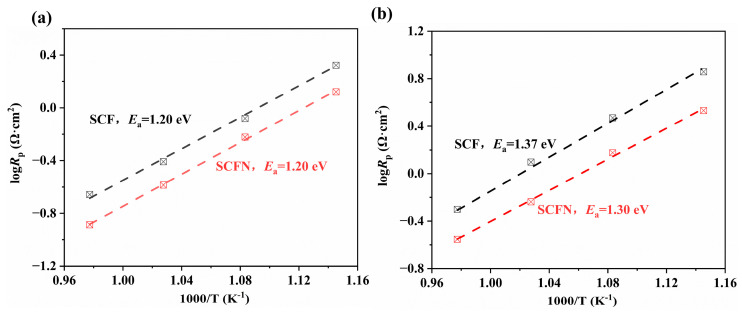
*E*_a_ of resistances for SCF and SCFN in air (**a**) and 5%H_2_ (**b**).

As shown in Figure 9a,b, the activation energies (*E*_a_) of polarization resistance for SCF and SCFN in air and 5%H_2_ were calculated by Arrhenius plots as follows:

(2) $$\log R_{p}=\log R_{0}-\frac{E_{a}}{2.303RT}$$

where *R*_o_ is the pre-exponential factor, *T* is the absolute temperature (K), and *R* is the molar gas constant (8.314 J mol^−1^K^−1^) [35]. In air, the *E*_a_ of polarization resistance for SCF is equal to the *E*_a_ for SCFN, which is 1.20 eV. This indicates that Ni-doping at the B-site has no impact on the *E*_a_ of polarization resistance. Instead, it only contributes to the gas transport and the formation of oxygen vacancies in the oxygen reduction reaction process. In 5%H_2_, the *E*_a_ of polarization resistance reduces via Ni-doping from 1.37 eV to 1.30 eV. That is, Ni-doping at the B-site is not only conducive to the gas transfer and oxidation reaction at the anode side, but also reduces the *E*_a_ of polarization resistance, which accelerates the hydrogen oxidation reaction of the interface between anode/electrolyte.

**Figure 10:**
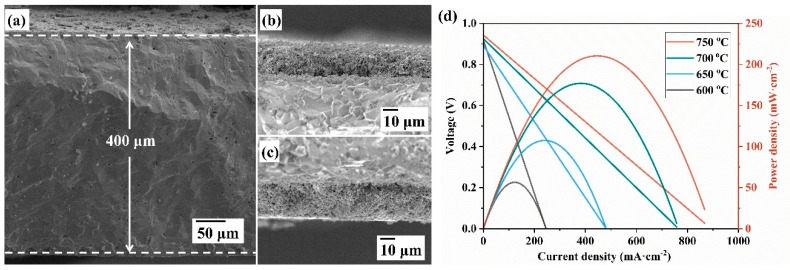
Cross-section views of cell SCFN-BZCY|BZCY|SCFN-BZCY. (**a**) Full view. (**b**) Detail view of interface between electrode and electrolyte. (**c**) Detail view of interface at other side. (**d**) I-V curves and power density of cell at various temperatures.

As shown in Figure 10a–c, the three-layer structure of the electrolyte supported symmetrical cell is composed of a porous cathode, dense electrolyte, and porous anode, with the thickness of electrolyte being 400 μm. To further assess the electrocatalytic activity of the symmetrical electrode, the symmetrical cell SCFN-BZCY|BZCY|SCFN-BZCY was operated in hydrogen and static air in Figure 10d. The open circuit voltage of the cell is at the range of 0.90–0.94 V, which indicates favorable gas tightness at the operating temperature. The current densities are 248.8, 484.2, 757.8, and 867.6 mA·cm^−2^ and the corresponding peak power densities are 56.5, 107.5, 177.0, and 210.4 mW·cm^−2^ at 600, 650, 700, and 750 °C. Considering the 400 μm thickness of the electrolyte derived from electrolyte supported symmetrical cell, the output performance is available, which is comparable to the SOFC with the Sr_0.8_Ce_0.2_FeO_3−δ_ symmetrical electrode (260.4 mW·cm^−2^, the thicken ss of electrolyte La_0.8_Sr_0.2_Ga_0.8_Mg_0.2_O_3−δ_ is 320 μm) [34]. It is worth noting that the more excellent performance may be realized by reducing the thickness of the electrolyte and optimizing the electrode technology, such as enhancing electrode performance by surface modification through the impregnation method, electro-spray deposition (ESD) [36], physical vapor deposition (PVD) [37,38], and atomic layer deposition (ALD) [39,40]. The best performance is 210.4 mW·cm^−2^ at 750 °C as the thickness of electrolyte BZCY is 400 μm, which illustrates that the material SCFN is supposed to be an excellent symmetrical electrode for PCFCs. The new finding provides a novel high efficiency symmetrical electrode material and guidance for achieving low-temperature PCFCs.

## 4. Conclusions

In summary, a novel symmetrical electrode of Ni-doped (Sr_0.8_Ce_0.2_)_0.95_FeO_3−δ_ is developed for PCFCs, with a porous honeycomb structure and matched thermal expansion coefficient with a proton-conducting electrolyte. Via Ni-doping at the B-site, (Sr_0.8_Ce_0.2_)_0.95_Fe_0.9_Ni_0.1_O_3−δ_ indicates reduced cell edge lengths, with the cell volume belonging to the space group *Pbnm* (62). The B-site elements in oxide tend to have a high oxidation state via Ni-doping, which is beneficial for forming more oxygen vacancies at high temperature and accelerating the cathodic oxygen reduction reaction. At the anode side, Ni-doping improves the stability of the symmetric electrode in the reducing atmosphere. The *R*_p_ of the symmetrical cell SCFN-BZCY|BZCY|SCFN-BZCY at 750 °C is 0.13 Ω cm^2^ and 0.28 Ω cm^2^, which is in air and 5%H_2_. Compared with the symmetrical cell of SCF, the *R*_p_ at 750 °C is 0.22 and 0.50 Ω cm^2^ in air and 5%H_2_, respectively. The *R*_p_ in the oxidation/reduction atmosphere reduced to half via Ni-doping. Meanwhile, Ni-doping reduces the *E*_a_ of impedance for the anode reaction in the symmetric cell. The peak power density of the symmetrical cell of SCFN is 210.4 mW·cm^−2^ at 750 °C. Since it is an electrolyte supported cell, electrochemical performance is already quite excellent considering the electrolyte thickness (400 μm), which demonstrated that Ni-doped SrFeO_3−δ_ is a promising symmetrical electrode for proton ceramic fuel cells, which is the guidance for the development of solid oxide fuel cells at reduced temperature.

## Figures and Tables

**Table 1:** The XRD refinement results of SCF and SCFN.

Samples	Cell Parameters (Å)			Cell Volume (Å^3^)	Reliability Factor (%)	
	a	b	c		*wR*_p_	*R*_p_
SCF	5.505	5.502	7.787	235.9	9.32	7.99
SCFN	5.491	5.502	7.768	234.7	9.89	8.11

**Table 2:** Atomic percents of SCF and SCFN before and after reduction reactions.

		O	Fe	Sr	Ce	Ni
SCF	Before	53.3	24.0	18.0	7.3	-
	After	48.8	29.2	14.3	5.4	-
SCFN	Before	53.7	21.1	18.3	4.5	2.5
	After	50.6	23.4	18.4	5.0	2.6

**Table 3:** The percentage compositions of the elements in SCF and SCFN before and after the reduction reactions.

Percentage Composition		SCF		SCFN	
		Before Reduction	After Reduction	Before Reduction	After Reduction
Fe	Fe^3+^	40.27	38.66	55.37	46.54
	Fe^2+^	59.73	61.34	44.63	53.46
O	O_lat_	49.59	51.48	53.80	55.14
	O_ads_	50.41	48.52	46.20	44.86
Ce	Ce^4+^	34.22	24.65	34.80	33.55
	Ce^3+^	65.78	75.35	65.20	66.45
Ni	Ni^3+^	-	-	48.81	32.08
	Ni^2+^	-	-	51.19	67.92

**Table 4:** The electrochemical impedance of the symmetrical cells in air and 5%H_2_.

T (°C)	SCF		SCFN	
	*R*_p_ in Air (Ω·cm^2^)	*R*_p_ in 5%H_2_ *R*_p_ (Ω·cm^2^)	*R*_p_ in Air (Ω·cm^2^)	*R*_p_ in 5%H_2_ *R*_p_ (Ω·cm^2^)
600	2.00	7.20	1.32	3.40
650	0.83	2.95	0.60	1.50
700	0.39	1.25	0.26	0.58
750	0.22	0.50	0.13	0.28

## Data Availability

The original contributions presented in this study are included in the article. Further inquiries can be directed to the corresponding author.

## Abbreviations

SOFCs	solid oxide fuel cells
PCFCs	proton ceramic fuel cells
XRD	X-ray diffraction
XPS	X-ray photoelectron spectroscopy
TGA	Thermogravimetric Analysis
TEC	thermal expansion coefficient
SEM	scanning electron microscopy
EDS	Energy Dispersive X-ray Spectroscopy
*R*_p_	polarization resistance
*R*_o_	Ohmic Resistance
*T*	temperature
Z′	The real part of the impedance spectrum
−Z″	The imaginary part of the impedance spectrum
*E*_a_	activation energy
